# Characterizing the Natural History of Pediatric Brain Tumors Presenting with Metastasis

**DOI:** 10.3390/cancers17050775

**Published:** 2025-02-24

**Authors:** Victor M. Lu, Toba N. Niazi

**Affiliations:** 1 Department of Neurological Surgery, Miller School of Medicine, University of Miami, Miami, FL 33136, USA; 2 Department of Neurological Surgery, Nicklaus Children’s Hospital, Miami, FL 33155, USA

**Keywords:** pediatric, brain tumor, CNS, metastasis, metastatic disease

## Abstract

Background: The natural history of pediatric patients with metastasis of primary brain tumors within and outside the central nervous system is poorly understood, as too are possible clinical correlates with outcome. Correspondingly, the aim of this study was to interrogate a national database to characterize this diagnosis and its clinical course in pediatric patients. Methods: The U.S. National Cancer Database (NCDB) was interrogated between the years 2005–2016 for all patients aged 18 years and younger with a primary brain tumor diagnosis, as well as evidence of disease metastasis at initial diagnosis. Data were summarized and overall survival (OS) was modeled using Kaplan–Meier and Cox regression analyses. Results: Out of a total of 8615 pediatric brain tumor patients, 356 (4%) had evidence of metastasis at initial diagnosis. Compared to patients without metastasis, patients with metastasis were statistically younger, more often male, and less likely to have private health insurance (all *p* < 0.050). With respect to clinical characteristics, the primary tumors of patients with metastasis were statistically more likely to be located in the cerebellum; be of higher histologic grading, with a higher proportion of medulloblastoma diagnoses and lower proportion of malignant glioma and pilocytic astrocytoma diagnoses; and were more likely to be treated by subtotal surgical resection, chemotherapy and radiation therapy (all *p* < 0.050) when compared to patients without metastasis. Five-year OS for those with metastasis was significantly lower than those without (48% vs. 75%, *p* < 0.001), with the median overall survival for patients with metastasis being 53 months (95% CI 29–86). Multivariate analysis indicated that a shorter OS was independently associated with the primary diagnoses of malignant glioma (HR 27.7, *p* = 0.020) and Atypical Teratoid/Rhabdoid Tumor (ATRT, HR 41.1, *p* = 0.041) and with WHO grades 3 (HR 20.1, *p* = 0.012) and 4 (HR 11.5, *p* < 0.001). Longer OS was significantly and independently associated with surgery (HR 0.49, *p* < 0.001), chemotherapy (HR 0.53, *p* = 0.041), and radiation therapy (HR 0.57, *p* = 0.026). Conclusions: Although uncommon, pediatric brain tumors with evidence of metastasis at initial diagnosis will present with a distinct socioeconomic and clinical profile compared to patients without metastasis. Multiple predictors are independently associated with overall prognosis, and understanding these features should be validated in prospective efforts to identify vulnerable patients earlier in order to maximize the impact of treatment.

## Simple Summary

Pediatric brain tumors are uncommon, and presentation with metastases either within or outside the brain and spine are even less common. As such, the natural history of these patients is poorly understood. We sought to interrogate a national database to create the largest cohort to date of these patients for analysis to better understand their natural history. We found that these patients with metastases had a distinct socioeconomic and clinical profile compared to patients without metastasis, including younger ages, a higher proportion of male individuals, a lower likelihood of having private insurance in terms of demographics, and shorter overall survival. These differences highlight the need to further study this population to improve targeted interventions and outcomes.

## 1. Introduction

Central Nervous System (CNS) tumors are the second most common tumor in pediatric patients after leukemia, the most common solid tumor in this demographic, and, collectively, they are the leading cause of cancer-related mortality overall in this age group [1,2]. Estimates suggest that in the United States alone, over 5000 new cases of CNS tumors are expected to be diagnosed in children and adolescents in 2023 [3]. Given these statistics, understanding how these patients present and what can vary at the time of presentation is important.

**Figure 1:**
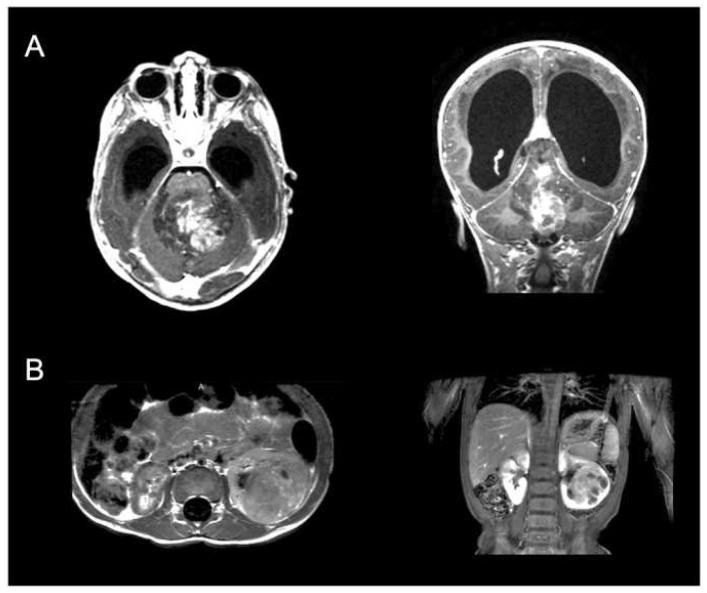
Example of a 15-month-old boy that presented with a 5-month history of spasticity in lowers, headaches, and vomiting. (**A**). MRI of the brain revealed a large fourth ventricular mass with associated hydrocephalus, and (**B**). MRI of the abdomen demonstrated left renal mass. Ultimately, the patient was taken to the operating room for decompression of the fourth ventricular mass without issue, and then, four weeks later, underwent a partial left nephrectomy. Pathology of the brain lesion revealed a diagnosis of Atypical Teratoid/Rhabdoid Tumor (ATRT) and similar histology for the renal lesion, indicating metastatic disease. The patient subsequently underwent adjuvant radiation therapy and chemotherapy. Two years later, the patient remains alive, having undergone two CSF diversion procedures, and continues to receive adjuvant treatment.

Although rare, one underexplored presentation is pediatric CNS tumors within the brain that present with metastasis away from the primary site, including the brain, bone, and solid organs such as the liver and kidney (Figure 1). What makes this particular cohort special is that the definition of metastasis in the setting of a primary brain tumor can include other distinct sites in the brain and spine, as well as other sites outside the CNS, which is more typical by definition [4,5]. Institutional series estimate that these metastases of pediatric CNS tumors will occur in approximately 0.5–2% of all presenting pediatric brain tumors, although more pathology-specific series indicate this could be as high as 40% [6,7,8,9].

Metastases upon presentation have been linked to poorer prognosis in different types of adult and pediatric brain tumors [10,11,12], but, to date, there has been no attempt to profile and characterize pediatric brain tumors presenting with metastasis to evaluate for survival correlates. Correspondingly, the aim of this study was to interrogate a national cancer database to establish a socioeconomic and clinical profile of pediatric brain tumors presenting with metastasis and to evaluate any clinical predictors of prognosis.

## 2. Methods

### 2.1. Patient Selection

All data used for this study were extracted from the 2016 iteration of the National Cancer Database (NCDB), a database maintained by the Commission on Cancer (CoC) and the American Cancer Society since 2004 that describes over 70% of new cancer diagnoses from 1500 hospitals in the U.S. [13] The database was queried for all patients that satisfied the following inclusion criteria: (1) a surgically proven (biopsy or resection) primary brain tumor diagnosis (defined by ICD-O-3.2 codes, Supplementary Table S1), (2) a confirmed presence of distant metastasis from the site of the primary lesion, (3) in patients aged ≤ 18 years old, and (4) having known treatment (surgical resection, chemotherapy and radiation therapy) statuses and overall survival outcomes. Exclusion criteria were (1) recurrent pathology, (2) non-intracranial primary lesions, (3) adult patients, and (4) incomplete clinical data.

### 2.2. Data and Outcomes

Demographic data were directly abstracted from the database listings. These data were age, sex, race, insurance status, yearly income, education level, metropolitan residence, and co-morbidity status. The primary outcome of interest was overall survival (OS). The presence of distant metastasis was defined using the ‘cs_mets_at_dx’ variable. Surgical resection was defined as any surgical resection listed in the ‘cs_sitespecific_factor_7’ variable. Chemotherapy was defined as patients receiving either single- or multiple-agent chemotherapy agent regimens as listed in the ‘rx_summ_chemo’ variable. Radiation therapy was defined as treatment by any beam radiation regimen as listed in the ‘rx_summ_radiation’ variable.

### 2.3. Statistical Analyses

Outcome comparisons between groups were conducted using the chi-square exact test and the Wilcoxon rank-sum test for categorical and continuous data, respectively. Kaplan–Meier estimations using log rank testing were used to compare overall survival (OS). For all regression analyses, univariable analyses were conducted first to identify candidate associations; based on these results, variables demonstrating a between-groups test statistic of *p* < 0.10 were included in multivariable analysis to determine the independence of these factors reported as hazard ratio (HR) with a 95% confidence interval (CI). All analyses were conducted using STATA 14.1 (StataCorp, College Station, TX, USA); tests were two-sided, and statistical significance was defined using the alpha threshold of 0.05.

## 3. Results

### 3.1. Demographics

A total of 8615 pediatric brain tumor patients satisfied all selection criteria, of which 356 (4%) had documented evidence of metastatic disease at diagnosis (Table 1). Overall, patients were diagnosed at a mean age of 8.8 years. There were more males (55%) in gender, with the racial majority being white (76%) and the minority Hispanic (18%). Private insurance was the most common (60%), the highest family income quartile was the most common (37%), there was an even distribution between average parent education level quartiles, and the majority of patients were based in a metropolitan setting (83%) without any co-morbidities (90%). Compared to those without metastases, patients with metastases were statistically diagnosed at a younger age (6.0 vs. 8.9 years, *p* < 0.001), more likely to be male (67% vs. 54%, *p* < 0.001), and less likely to have private insurance (50% vs. 61%, *p* = 0.001).

### 3.2. Clinical Features

The clinical features of the primary brain tumor were then characterized (Table 2). Overall, the most common locations were the cerebellum (19%) and brainstem (18%), and the most common WHO grades were 1 (40%) and 4 (30%), with malignant glioma (20%), pilocytic astrocytoma (19%), and medulloblastoma (13%) amongst the more common diagnoses. Compared to those without metastases, the primary tumors of patients with metastases were statistically more likely to be located in the cerebellum (34% vs. 18%, *p* < 0.001), be WHO grade 4 (75% vs. 28%, *p* = 0.001), and have a higher proportion of medulloblastoma diagnoses (46% vs. 12%, *p* < 0.001). Common sites of metastasis included the brain (17%) and bone (10%).

### 3.3. Treatment Features

Overall, the majority of patients underwent surgical resection for their primary tumors (75%) and were not treated with chemotherapy (65%) or radiation therapy (64%) (Table 2). However, compared to those without metastasis, the primary tumors of patients with metastases were statistically more likely to have had subtotal resection (28% vs. 17%, *p* < 0.001) and been treated by both chemotherapy (77% vs. 34%, *p* < 0.001) and radiation therapy (60% vs. 35%, *p* < 0.001).

### 3.4. Overall Survival and Predictors

**Figure 2:**
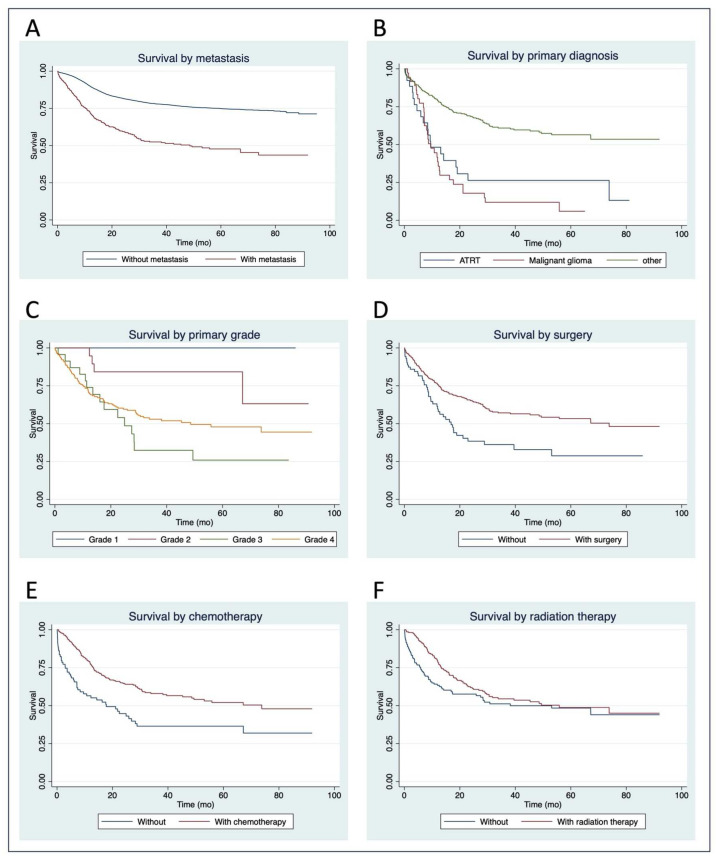
A series of Kaplan–Meier curves indicating survival trends in the included cohort (**A**). by presence of metastasis, (**B**). by primary diagnosis, (**C**). by primary tumor grade, (**D**). by surgery, (**E**). by chemotherapy, and (**F**). by radiation therapy.

Five-year OS for those with metastasis was significantly lower than those without (48% vs. 75%, *p* < 0.001) (Figure 2A), with the median overall survival of patients with metastasis being 53 months (95% CI 29–86). All reported variables in Table 1 and Table 2 were tested using a univariate regression analysis. Multiple candidate parameters were identified within the primary site: primary WHO grade, histology, and treatment variables. Multivariate analysis indicated that shorter OS was significantly and independently associated with primary diagnoses of malignant glioma (HR 27.7, 95% CI 1.70–451, *p* = 0.020) and Atypical Teratoid/Rhabdoid Tumor (ATRT, HR 41.1, 95% CI 3.11–267, *p* = 0.041) when compared to pilocytic astrocytoma diagnosis, and with WHO grades 3 (HR 20.1, 95% CI 1.95–207, *p* = 0.012) and 4 (HR 11.5, 95% CI 1.10–120, *p* < 0.001) when compared to WHO grade 1 (Figure 2B,C). Longer OS was significantly and independently associated with surgery (HR 0.49, 95% CI 0.41–0.59, *p* < 0.001), chemotherapy (HR 0.53, 95% CI 0.28–0.97, *p* = 0.041), and radiation therapy (HR 0.57, 95% CI 0.35–0.94, *p* = 0.026) (Figure 2D–F).

## 4. Discussion

The natural history of pediatric brain tumor patients with evidence of metastases at initial diagnosis is not well defined because primary brain tumors rarely metastasize to other sites in the CNS as well as other sites outside the CNS. Our study demonstrates that these patients are younger than those without metastases, along with other defining differences, and portend to a significantly shorter OS compared to those without metastasis. More pertinent to the primary pathology, multiple predictive parameters of OS appear contingent on the clinical characteristics of the primary brain tumor. The pediatric population is different in terms of ATRT and other pathology prevalences, as well as tolerance of different adjuvant therapies, highlighting that this specific niche requires its own attention rather than relying solely on extrapolation from adult experiences.

The prototypic pediatric brain tumor patient with metastasis is not well-defined. To date, there is no scoping summary across all pediatric brain tumors, but anecdotal literature confirms components of our results. A survey of the literature by Rickert [14] found that the majority of pediatric cases of extraneural metastases from primary brain tumors occurred in males, with medulloblastoma being the most common primary diagnosis. In a series of medulloblastomas with metastatic spread at initial diagnosis by Zapotocky et al. [6], the most common location for the primary lesion was the posterior fossa. This is not necessarily surprising, given the predilection this tumor has for the cerebellum and fourth ventricle. In their series of ATRT, Yamasaki et al. [15] reported that those patients with metastatic disease were treated with higher rates of chemotherapy and radiation therapy than those without. Finally, Yang et al. [16], in their series evaluating leptomeningeal metastasis secondary to gliomas in adults, found that a higher WHO grade was independently associated with higher rates of metastasis, which intuitively aligns with our observations.

Outside the predictive significance of primary grade and adjuvant chemoradiation therapies found through multivariate regression analysis, there were absences that highlight the difference of this target diagnosis compared to more general brain tumor cohorts. For example, no socioeconomic parameter was independently predictive of outcome. Various database studies for more generalized pediatric tumor groups, such as all medulloblastoma [17] general pediatric cancers [18], have highlighted older age, female gender, and insurance coverage to predict better OS. Given the lack of any of these associations, it suggests that this specific presentation in question may possibly be independent of socioeconomic parameters due to a more aggressive natural history [8].

Another aspect to consider is the fact that surgical resection of the primary tumor did impact OS. Multiple examples exist in the literature where the extent of the resection of pediatric brain tumors in general impacts OS in the absence of metastasis at diagnosis, such as ependymoma [19] and low-grade glioma [20]. We note, however, that in more specific cohorts of higher-grade CNS tumors, such as choroid plexus carcinoma [21] and ATRT [22], surgical resection does not impact OS. This therefore posits that pediatric brain tumor diagnoses that present with metastasis may be more malignant in nature than an isolated primary diagnosis. Surgically, it reasons that single intracranial lesions are more amenable to treatment than diffuse disease, but their surgical resection still affords some clinical benefit.

Moving forward, it is likely that progress in managing these patients will be made specific to the primary pathology in question. This will be because of greater molecular understanding of different primary and metastasis subtypes [23], as well as what interventions are available and effective based on the age of typical presentation. For example, cases of medulloblastoma presenting with metastasis at initial diagnosis have been shown to be subtype-dependent [6]. Groups 3 and 4 have greater metastatic potential than Wnt and SHH subgroups, and even within Groups 3 and 4, laminar metastases were more common in Group 3, while nodular and suprasellar metastases were more common in Group 4. Another example to consider is the incidence of pilocytic astrocytomas presenting with metastases. Typically considered benign WHO Grade 1 lesions, the database showed that this pathologic diagnosis can also present with metastases too, an observation that has been reported in case reports [24,25]. This presents an interesting discordance between histologic grading and pathologic diagnosis, considering that the presence of metastasis implies a histology more severe than the pathology typically is. Therefore, a greater understanding of the molecular make-up of the primary tumor, either benign or malignant, will be pivotal in understanding this rare phenomenon of metastasis.

There are limitations to consider. The first is the heterogeneous nature of the pediatric brain tumors that are included in this scoping study, despite the relatively low number of cases needed to create statistical power. Specific diagnoses, such as ATRT, medulloblastoma, and even malignant gliomas of the brainstem, are often clinically and molecularly distinct, and, moving forward, their treatments even in the presence of metastasis will be distinct as well [26]. For example, brainstem gliomas are typically considered not surgical [27], so their inclusion into this cohort regression analysis evaluating surgical resection will bias the result towards the null hypothesis.

Another example is that, since the introduction of the latest WHO Classification [28], brain tumors now often require a molecular component to their diagnosis, e.g., medulloblastoma Group 3 versus Wnt Group. However, the NCDB data precedes the latest classification system and, as such, does not provide this data. Although our results are robust with regard to the classic WHO histological grading, future studies focused on molecular and histologic subtyping will enhance our understanding further of how our results fit in the molecular era. This will be even applicable to the setting of glioma origin, be it astrocytic or oligodendroglic in nature, given that across different WHO gradings, their baseline prognoses are known to be different [29]. Although not available in the NCDB, prospective data such as this will greatly enhance the potential of understanding these metastases, too.

Our findings are also affected by the limited details regarding the metastases that define our group of interest. Although the NCDB is able to provide some degree of specificity by including parameters specific to other brain and bone sites of metastasis, it does not have the granularity to distinguish all solid organ targets, and neither can it highlight specific sites within the neuro-axis, such as the leptomeninges versus the drop metastases within the spine [30]. This is important; for example, an isolated solid organ lesion amenable to gross total resection will be surgically triaged very differently to diffuse leptomeningeal spread. Furthermore, this is crucial because the nature of the metastases themselves are important considerations, such as one metastasis within the CNS versus multiple metastases outside the CNS versus overlapping metastases. A dedicated prospective data collection effort is likely the most effective way to obtain this information in the future.

Finally, there is an understanding that our results may underestimate the true nature of pediatric brain tumors with metastasis at initial diagnosis; this is primarily because, in order to be precise, all 8615 patients would have presumably been screened for all possible metastatic sites at initial diagnosis, which cannot be confirmed retrospectively. Patients with less insurance coverage, lower income, and less medical awareness who present themselves to small, rural facilities may not have the resources and abilities to conduct such evaluations. For example, dedicated tertiary academic pediatric centers report synchronous metastatic disease at initial presentation for medulloblastoma [6] and ATRT [8] in 23% and 38% of patients, respectively, which compares to 15% and 12% based on our NCDB experience. We suspect that, as awareness of the metastatic potential of primary pediatric brain tumors continues to grow, this will lead to higher rates of metastasis surveillance in the future.

## 5. Conclusions

There is a subset of pediatric primary brain tumor patients that will present with metastatic spread of their disease elsewhere in the brain, spine, and body. Our study shows that, at initial evaluation, these patients have a median overall survival of 53 months, which is statistically shorter than those patients without metastasis. These patients have a distinct socioeconomic and clinical profile that separates them from patients without metastasis. These include younger patients without private health insurance, two sociodemographic parameters that health organizations can better analyze to generate more targeted policies to improve this disparity.

Multiple predictors of prognosis are contingent on the primary diagnosis, and, as such, moving forward, treatment and innovation will need to be diagnosis- and age-specific, given the large heterogeneity in pediatric brain tumors in general. Furthermore, our study demonstrates that primary treatment of the primary brain tumor can result in favorable overall outcomes in the presence of these metastases, meaning that further initiatives to augment the availability of all treatment options should be the focus of future studies.

## Figures and Tables

**Table 1:** Demographic features of the overall cohort. Continuous data presented as mean ± SD, and categorical data presented as n (% total).

	All Patients (n = 8615)	Metastasis Present (n = 356)	Metastasis Absent (n = 8259)	*p*
Demographic features				
Age (years)	8.8 ± 5.5	6.0 ± 4.6	8.9 ± 5.5	<0.001
Gender				<0.001
Female	3901 (45%)	117 (33%)	3784 (46%)	
Male	4714 (55%)	239 (67%)	4475 (54%)	
Race				0.054
White	6573 (76%)	266 (75%)	6306 (76%)	
Black	1115 (13%)	38 (11%)	1077 (13%)	
Other	927 (11%)	52 (14%)	876 (11%)	
Hispanic status				0.098
Hispanic	1555 (18%)	76 (21%)	1479 (18%)	
Non-Hispanic	7060 (82%)	280 (79%)	6780 (82%)	
Insurance status				0.001
Private	5177 (60%)	178 (50%)	4999 (61%)	
Medicaid/Medicare	2915 (34%)	154 (43%)	2761 (33%)	
Other	333 (4%)	16 (4%)	317 (4%)	
None	190 (2%)	8 (2%)	182 (2%)	
Family annual income				0.052
>$63,333	3177 (37%)	110 (31%)	3067 (37%)	
$50,354–$63,332	1958 (23%)	97 (28%)	1861 (23%)	
$40,227–$50,353	1836 (22%)	72 (21%)	1764 (22%)	
<$40,227	1565 (18%)	71 (20%)	1494 (18%)	
Parent education level				0.134
<6.3%	2124 (25%)	72 (20%)	2052 (25%)	
6.3–10.8%	2234 (26%)	88 (25%)	2146 (26%)	
10.9–17.5%	2113 (25%)	100 (28%)	2013 (25%)	
>17.6%	2075 (24%)	92 (26%)	1983 (24%)	
Residence				0.266
Metropolitan	7179 (83%)	289 (81%)	6890 (83%)	
Urban/rural	1436 (17%)	167 (19%)	1369 (16%)	
Co-morbidity				0.207
Yes	872 (10%)	29 (8%)	843 (10%)	
No	7743 (90%)	327 (92%)	7416 (90%)	

**Table 2:** Clinical and treatment features of the overall cohort. Categorical data presented as n (% total). WHO, World Health Organization; ATRT, atypical teratoid rhabdoid tumor.

	All Patients (n = 8615)	Metastasis Present (n = 356)	Metastasis Absent (n = 8259)	*p*
Clinical features				
Primary site				<0.001
Cerebrum	709 (8%)	20 (6%)	689 (8%)	
Frontal lobe	768 (9%)	7 (2%)	761 (9%)	
Temporal lobe	817 (10%)	14 (4%)	803 (10%)	
Parietal lobe	356 (4%)	5 (1%)	351 (4%)	
Occipital lobe	185 (2%)	1 (<1%)	184 (2%)	
Ventricle	733 (8%)	34 (10%)	699 (9%)	
Cerebellum	1608 (19%)	121 (34%)	1487 (18%)	
Brain stem	1499 (17%)	61 (17%)	1438 (17%)	
Overlapping lesion	365 (4%)	12 (3%)	353 (4%)	
Not specified	1575 (18%)	81 (23%)	1494 (18%)	
Primary WHO grade *				<0.001
1	2120 (40%)	15 (6%)	2105 (41%)	
2	868 (16%)	20 (8%)	848 (17%)	
3	720 (13%)	23 (10%)	697 (14%)	
4	1616 (30%)	178 (75%)	1438 (28%)	
Histology **				<0.001
Medulloblastoma	1118 (13%)	164 (46%)	954 (12%)	
Malignant glioma	1682 (20%)	40 (11%)	1642 (20%)	
ATRT	211 (2%)	26 (7%)	185 (2%)	
Pilocytic astrocytoma	1643 (19%)	24 (7%)	1819 (20%)	
Ependymoma	612 (7%)	19 (5%)	593 (7%)	
Neuroblastoma	26 (<1%)	13 (4%)	13 (<1%)	
Primary site of metastasis ***				
Brain		60 (62%)		
Bone		37 (38%)		
Treatment features				
Surgery				<0.001
No, autopsy only	2120 (25%)	73 (21%)	2047 (25%)	
Yes				
Biopsy	1437 (17%)	63 (18%)	1374 (17%)	
Subtotal resection	1499 (17%)	100 (28%)	1399 (17%)	
Gross total resection	3559 (41%)	120 (34%)	3439 (42%)	
Chemotherapy				<0.001
Yes	3044 (35%)	275 (77%)	2769 (34%)	
No	5571 (65%)	81 (23%)	5490 (66%)	
Radiation therapy				<0.001
Yes	3127 (36%)	212 (60%)	2915 (35%)	
No	5488 (64%)	144 (40%)	5322 (65%)	

* All patients n = 5324, metastasis present n = 236, metastasis absent n = 5088. ** Histology listed only where percentage in the group with metastasis > 2%. *** Only n = 97 patients reported metastases with a defined site. The metastases site for all other patients not listed.

## Data Availability

Available upon request.

## Supplementary Materials

The following supporting information can be downloaded at: https://www.mdpi.com/article/10.3390/cancers17050775/s1, Table S1: Corresponding ICD-0-3 SEER Site/Histology Codes used.
