# Supplementary material for: Characterizing the Natural History of Pediatric Brain Tumors Presenting with Metastasis
Source: Cancers (Basel). 2025 Feb 24;17(5):775. doi: 10.3390/cancers17050775 (PMC11898785; doi:10.3390/cancers17050775)
Supplement: Supplementary file 1 [file cancers-17-00775-s001.zip › cancers-3450917-supplementary.pdf]

Supplementary Table S1. Corresponding ICD-0-3 SEER Site/Histology Codes used.

8000/0 Neoplasm, benign
8000/1 Neoplasm, uncertain whether benign or malignant
8000/3 Neoplasm, malignant
8001/0 Tumor cells, benign
8001/1 Tumor cells, uncertain whether benign or malignant
8001/3 Tumor cells, malignant
8002/3 Malignant tumor, small cell type
8003/3 Malignant tumor, giant cell type
8004/3 Malignant tumor, spindle cell type
8005/3 Malignant tumor, clear cell type
8680/1 Paraganglioma, NOS
8720/3 Malignant melanoma, NOS
8800/0 Soft tissue tumor, benign
8800/3 Sarcoma, NOS
8801/3 Spindle cell sarcoma
8805/3 Undifferentiated sarcoma
8806/3 Desmoplastic small round cell tumor
8815/1 Solitary fibrous tumor/hemangiopericytoma Grade 2
8850/0 Lipoma, NOS
8851/0 Fibrolipoma
8851/3 Liposarcoma, well differentiated
9060/3 Dysgerminoma
9064/3 Germinoma
9070/3 Embryonal carcinoma, NOS
9071/3 Yolk sac tumor
9080/0 Teratoma, benign
9080/1 Teratoma, NOS
9080/3 Teratoma, malignant, NOS
9081/3 Teratocarcinoma
9085/3 Mixed germ cell tumor
9100/3 Choriocarcinoma
9120/0 Hemangioma, NOS
9121/0 Cavernous hemangioma
9122/0 Venous hemangioma
9131/0 Capillary hemangioma
9150/1 Hemangiopericytoma, NOS
9161/1 Hemangioblastoma
9370/3 Chordoma, NOS
9371/3 Chondroid chordoma
9372/3 Dedifferentiated chordoma
9380/3 Glioma, malignant
9381/3 Gliomatosis cerebri
9382/3 Mixed glioma
9383/1 Subependymoma
9384/1 Supependymal giant cell astrocytoma
9385/3 Diffuse midline glioma, H3 K27M-mutant
9391/3 Ependymoma, NOS
9392/3 Ependymoma, anaplastic
9393/3 Papillary ependymoma
9394/1 Myxopapillary ependymoma
9396/3 Ependymoma, RELA fusion-positive
9400/3 Astrocytoma, NOS
9401/3 Astrocytoma, anaplastic
9410/3 Protoplasmic astrocytoma
9411/3 Gemistocytic astrocytoma
9412/1 Desmoplastic infantile astrocytoma
9413/0 Dysembryoplastic neuroepithelial tumor

9420/3 Fibrillary astrocytoma
9421/1 Pilocytic astrocytoma
9421/3 Pilocytic astrocytoma
9423/3 Polar spongioblastoma
9424/3 Pleomorphic xanthoastrocytoma
9425/3 Pilomyxoid astrocytoma
9430/3 Astroblastoma
9431/1 Angiocentric glioma
9440/3 Glioblastoma, NOS
9441/3 Giant cell glioblastoma
9442/1 Gliofibroma
9442/3 Gliosarcoma
9444/1 Chordoid glioma
9445/3 Glioblastoma, IDH-mutant
9450/3 Oligodendroglioma, NOS
9451/3 Oligodendroglioma, anaplastic
9460/3 Oligodendroblastoma
9473/3 Primitive neuroectodermal tumor
9475/3 Medulloblastoma, WNT-activated
9476/3 Medulloblastoma, SHH-activated and TP53-mutant
9477/3 Medulloblastoma, non-WNT/non-SHH
9478/3 Embryonal tumor with multilayered rosettes, NOS
9490/0 Ganglioneuroma
9490/3 Ganglioneuroblastoma
9492/0 Gangliocytoma
9500/3 Neuroblastoma, NOS
9501/3 Medulloepithelioma, NOS
9502/3 Teratoid medulloepithelioma
9503/3 Neuroepithelioma, NOS
9505/1 Ganglioglioma, NOS
9505/3 Ganglioglioma, anaplastic
9508/3 Atypical teratoid/rhabdoid tumor
9509/1 Papillary glioneuronal tumor
9540/0 Neurofibroma, NOS
9540/1 Neurofibromatosis, NOS
9540/3 Malignant peripheral nerve sheath tumor
9541/0 Melanotic neurofibroma
9550/0 Plexiform neurofibroma
9560/0 Neurilemoma, NOS
9560/1 Melanotic schwannoma
9560/3 Neurilemmoma, malignant
9561/3 MPNST with rhabdomyoblastic differentiation
9562/0 Neurothekeoma
9570/0 Neuroma, NOS
9571/0 Perineurioma, NOS
9571/3 Perineurioma, malignant
9590/3 Malignant lymphoma, NOS
9591/3 Malignant lymphoma, non-Hodgkin
9596/3 Composite Hodgkin and non-Hodgkin lymphoma
9670/3 ML, small B lymphocytic, NOS
9671/3 ML, lymphoplasmacytic
9673/3 Mantle cell lymphoma
9675/3 ML, mixed sm. and lg. cell, diffuse
9680/3 ML, large B-cell, diffuse
9684/3 ML, large B-cell, diffuse, immunoblastic, NOS
9687/3 Burkitt lymphoma, NOS
9688/3 T-cell histiocyte rich large B-cell lymphoma
9690/3 Follicular lymphoma, NOS
9691/3 Follicular lymphoma, grade 2

9695/3 Follicular lymphoma, grade 1
9698/3 Follicular lymphoma, grade 3
9699/3 Marginal zone B-cell lymphoma, NOS
9701/3 Sezary syndrome
9702/3 Mature T-cell lymphoma, NOS
9705/3 Angioimmunoblastic T-cell lymphoma
9712/3 Intravascular large B-cell lymphoma
9714/3 Anaplastic large cell lymphoma, T-cell and Null cell type
9719/3 NK/T-cell lymphoma, nasal and nasal-type
9724/3 SystemicEBV pos. T-cell lymphoproliferative disease of childhood
9727/3 Precursor cell lymphoblastic lymphoma, NOS
9728/3 Precursor B-cell lymphoblastic lymphoma
9729/3 Precursor T-cell lymphoblastic lymphoma
9731/3 Plasmacytoma, NOS
9734/3 Plasmacytoma, extramedullary
9735/3 Plasmablastic lymphoma
9737/3 ALK positive large B-cell lymphoma
9738/3 Lrg. B-cell lymphoma in HHV8-assoc. multicentric Castleman DZ
9750/3 Malignant histiocytosis
9751/3 Langerhans cell histiocytosis, NOS
9754/3 Langerhans cell histiocytosis, disseminated
9755/3 Histiocytic sarcoma
9756/3 Langerhans cell sarcoma
9757/3 Interdigitating dendritic cell sarcoma
9758/3 Follicular dendritic cell sarcoma
9759/3 Fibroblastic reticular cell tumor
9811/3 B lymphoblastic leukemia/lymphoma, NOS
9812/3 Leukemia/lymphoma with t(9;22)(q34;q11.2);BCR-ABL1
9813/3 Leukemia/lymphoma with t(v;11q23);MLL rearranged
9814/3 Leukemia/lymphoma with t(12;21)(p13;q22);TEL-AML1(ETV6-RUNX1)
9815/3 B lymphoblastic leukemia/lymphoma with hyperdiploidy
9816/3 Leukemia/lymphoma with hypodiploidy (hypodiploid ALL)
9817/3 B lymphblastic leukemia/lymphoma with t(5;14)(q31;q32);IL3-IGH
9818/3 Leukemia/lymphoma with t(1;19)(q23;p13.3); E2A PBX1 (TCF3 PBX1)
9823/3 Chronic lymphocytic leukemia/small lymphocytic lymphoma
9831/3 T-cell large granular lymphocytic leukemia
9837/3 T lymphoblastic leukemia/lymphoma
9930/3 Myeloid sarcoma
9965/3 Myeloid and lymphoid neoplasms with PDGFRB rearrangement
9967/3 Myeloid and lymphoid neoplasm with FGFR1 abnormalities
9971/3 Polymorphic PTLD
9975/3 Myelodysplastic/Myeloproliferative neoplasm, unclassifiable
